# Cross-sectional assessment of perception and attitude of pharmacy students towards pharmaceutical promotion: a study from developing country, Pakistan

**DOI:** 10.3389/fmed.2024.1424352

**Published:** 2024-11-01

**Authors:** Ali Hassan Gillani, Hafsa Arshad, Muhammad Arshed, Ammar Jairoun, Sundus Shukar, Jamshaid Akbar, Azzah Khadim Hussain, Hafiz Muhammad Abbas Malik, Mohamed Izham Mohamed Ibrahim, Yu Fang

**Affiliations:** ^1^Department of Pharmacy Administration and Clinical Pharmacy, School of Pharmacy Xi’an Jiaotong University, Xi’an, Shaanxi, China; ^2^Center for Drug Safety and Policy Research, Xian Jiaotong University, Xi’an, Shaanxi, China; ^3^Shaanxi Center for Health Reform and Development Research, Xi’an, China; ^4^University Institute of Public Health Faculty of Allied Health Sciences, The University of Lahore, Lahore, Pakistan; ^5^Department of Pharmacy and Alternative Medicine, Islamia University of Bahawalpur, Bahawalpur, Pakistan; ^6^Department of Pharmacy, University of Central Punjab, Lahore, Pakistan; ^7^Department of Clinical and Pharmacy Practice, College of Pharmacy, QU Health, Qatar University, Doha, Qatar

**Keywords:** pharmaceutical promotion, pharmacy students, perception, attitude, Pakistan

## Abstract

**Background:**

The pharmaceutical promotion is “all informational and persuasive activities by manufacturers and distributors, the effect of which is to induce the prescription, supply, purchase and/or use of medicinal drugs. These promotional activities affect the dispensing behavior of physicians and pharmacists and influence begins from educational institutes.

**Objective:**

Our study’s main aim was to evaluate opinion and attitude of pharmacy students towards pharmaceutical promotion.

**Materials and methods:**

A cross-sectional online survey was conducted among 3rd, 4th and final year pharmacy students of 3 public and 3 private sector universities in Punjab Pakistan. A modified version of pre available questionnaire was used to collect data from students between June 2020 and December 2020. The tool was made available through a Google Form, assessable to students via provided link. We utilized the snowball sampling technique. Descriptive statistics were used to evaluate the demographics, while Chi-square and t-test were used to analyze associations between demographics and items. Data were analyzed using SPSS version 21.0.

**Results:**

A total of 1,195 students participated in the survey with an average age of 22.2 ± 1.2 years. Nearly 2/3rd of the students were males (62.2%) and a significant proportion (87.3%) of pharmacy students had never taken part in any training provided by pharmaceutical company. Among all, 51.9% confirmed that pharmacists who frequently interact with medical representatives tend to dispense more antibiotics. Additionally, 42.1% indicated they may dispense antibiotics under the influence of promotion. Pharmacy students in senior college years and those with lower parental exhibited significantly more perception and attitude scores (*p* < 0.001).

**Conclusion:**

A significant number of students concurred with the notion that promotional activities could impact dispensing practices and they also believed that such activities contribute to the growing issue of irrational antibiotic use. This study underscores the necessity for a heightened emphasis on the educational needs of pharmacy students.

## Introduction

Pharmaceutical promotions encompass a range of persuasive tactics employed by pharmaceutical companies to influence healthcare professionals including doctors and pharmacists to prefer their medications over those of other companies (1). While these promotions are essential for keeping medical professionals informed about drugs, they also play a significant role in reshaping prescribing and dispensing behaviors (2, 3). Evidence suggests that physician who are more receptive to gifts and interactions with medical representatives are more likely to prescribe the medications promoted by these representatives (4). Additionally, it has been reported that biased and misleading evidence presented by pharmaceutical companies may result in improper prescribing practices (5). Therefore, it becomes the responsibility of healthcare professionals to ensure that the information provided by medical representatives is impartial and not solely driven to make profit or intended to mislead decisions (6).

Students are an integral part of the general population given their influential decisions and the future positions they will hold within the community (7). They are under the continuous radar of pharmaceutical companies which are aiming to foster positive behaviors and a sense of obligation towards pharmaceutical companies and their products (4). It raises serious concerns about the potential influence of pharmaceutical promotions on medical and pharmacy students (8, 9). A study conducted at the clinical and preclinical students in United States of America reported that a large portion of respondents had participated in some form of promotional activity (10) and a study on 3rd years students indicated that despite being aware of the negative impacts of pharmaceutical promotion, all of them accepted the gifts. This suggests that students are influenced by pharmaceutical promotion early in their educational careers (11). In Pakistan Siddique UT and colleagues mentioned in their study that 81% of medical students expressed a preference for pharmaceutical company sponsored event or seminar at the college and more than two third were comfortable with receiving gifts from these companies (12). This conclusion highlights their high level of gift acceptability and emphasized on the need for incorporating guidelines into the medical curriculum.

Although pharmacists play a vital role in delivering proper education on diseases, drug selection and effective management, interactions between pharmacists-industry were perceived as less concerning compared to physician-industry relations (4, 6). Pharmacists occupy a significant role in discussing health policy matters and concerns related to medication therapy delivery for patients. They are also healthcare professionals who maintain close contact with pharmaceutical manufacturers (13). In Pakistan, due to lack of legislation and proper policy implementation, medications and antibiotics are available over the counter without prescriptions. Promotional tactics serve as a significant factor driving irrational antibiotics use (3). Antimicrobial resistance has been declared a public health emergency by the World Health Organization. Lower middle-income countries such as Nepal, Bangladesh, Pakistan, India, Cambodia, Thailand, Laos and Vietnam are particularly susceptible to antibiotic ineffectiveness due to absence of legislation, negligence by drug regulatory bodies, and the influence of promotional tactics (14). The persuasive pressure of promotion is greatly influencing physician’s prescribing pattern and contributing to the increased irrational prescription of antimicrobials (15). Due to their substantial influence in drug dispensing, pharmacists hold significant value for marketers and may be targeted more frequently at the college level (6, 16).

Despite all the facts mentioned above pharmacy students are not given the same level of attention in Pakistan and globally as medical students. Therefore, a cross-sectional survey was conducted among pharmacy students at 6 universities in Punjab, Pakistan. The aim was to determine students’ exposure to pharmaceutical promotion, their attitudes towards and acceptance of industry marketing strategies and gifts. We also assessed whether these attitudes were influencing future medicine dispensing practices particularly antibiotics.

## Materials and methods

### Study design and setting

Pakistan comprises of four provinces (Punjab, Baluchistan, Sind and Khyber Pakhtunkhwa) and two independent administrative territories (Gilgit Baltistan and Azad Jammu Kashmir). Although, Punjab covers 1/4th (26%) of Pakistan’s’ total land area, it is the most populous of all provinces accounting for 60% of the total population (17). We conducted a cross-sectional study in 6 universities of Punjab, Pakistan (three public sector and 3 private sector universities). Each of these universities was selected randomly and had an average batch size of 100 students per class. The pharmacy program (PharmD), titled Doctor of Pharmacy is a five-year bachelor’s degree, with clinical rounds in hospital wards during year 4 and 5. Students were also accustomed to complete clinical research projects. Some students received training on community pharmacy structures as part of the pharmacy curriculum. A few students chose to pursue practice placements in hospital pharmacies and government polyclinics immediately after their final year exams.

### Study population and data collection

All female and male pharmacy students, who were currently enrolled in 3rd, 4th or 5th year of study at universities were targeted in the survey. We conducted online survey due to partial and complete lockdowns during the COVID-19 outbreak. The online platform was chosen for data acquisition because conducting a community-based survey during this time was challenging. In Pakistan, a large proportion (76 million) of people frequently use the internet, with 37 million actively engaging on various social media platforms (18). We employed Snowball sampling and the survey tool was made accessible on Google forms from June 2020 to December 2020. We contacted student from the selected universities via phone with the assistance of their class administrators. Subsequently, a few students were provided with the survey link and instructed to share it with their peers. It was advised to share with 3rd fourth and final year students. The link contained a brief research introduction, information about confidentiality, consent, voluntary participation and the right to withdraw, on the first page. By clicking the link, participants could access the content of the study questionnaire and respond to questions. Participation was voluntary and no incentives were provided. To boost survey participation a reminder was sent to student after 2 weeks of survey initiation. Survey response were collected anonymously relying on honor system, trusting that students would complete the questionnaire only once and students were advised to fill it once. Medical representatives or medical sales representatives promote and sell medical products, including equipment, prescription medicines and drugs manufactured by their company to different healthcare facilities. They ensure that a medical facility has the proper medical supplies to operate and serve its patients.

### Study instrument

We conducted a comprehensive literature search on PubMed, google scholar and google using keywords such as “perception,” “attitudes,” “pharmacy students,” “pharmaceutical industry,” “education” and “Pakistan” in various combinations. This was done to identify relevant studies both from Pakistan and globally. To the best of our ability, we found only few studies on the presented topic (4, 6, 12, 19). Numerous studies were available regarding the medical student’s interaction with pharmaceutical companies around the globe and in Pakistan (8–12, 20). This apparent gap promoted the initiation of such a study in Pakistan. Consequently, a detailed and systematic review of related research and guidelines was conducted and a previously developed questions were taken from different data tools (4, 6, 12, 19). A comprehensive 61-item tool was constructed using available data and many questions were adapted from the survey tool used for the medical students, and these were subsequently modified for pharmacy students. The questionnaire sought information in 4 parts 1: demographic details of pharmacy student (14items), 2: pharmacy students’ perceptions of pharmaceutical promotion (19 Items), 3: attitudes/behaviors of pharmacy students towards promotion, policies or guidelines regarding interactions with the pharmaceutical companies, curriculum adequacy and the impact of pharmaceutical promotion on future drug and antibiotic dispensing (24 items). The term attitude was defined according to the oxford dictionary as; ‘a settled way of thinking or feeling reflected in a person’s behavior’ whereas in sociology attitude is written as orientation (towards a person, situation, institution, or social process) which is indicative of an underlying value or belief. Section 4: included a single question about the acceptance of valuable gifts, scenario of fee acceptance, a statement about medical representatives and 2 open ended questions. The detailed questionnaire is in Supplementary material S1. Only the principal investigator had access to the google account and the data analysis file was directly imported from the site. All items in section 2 (perception) and section 3 (attitude) were evaluated using a 5-point Likert scale (5 = Strongly Agree, 4 = agree, 3 = Neutral, 2 = disagree and 1 = strongly disagree).

Once, the tool’s conceptualization was completed, its content validity was assessed by two professors with a background in pharmacy practice. Minor adjustments were made to the questionnaire for our study including the conversion of USD ($) to Pakistani Rupee (PKR) for parental income, and the addition of two open-ended questions at the end. These additional questions aimed to gather detailed information about individuals’ interactions with medical representatives as investigating the issue required comprehensive understanding of student-medical representatives’ interactions. This modified version was then pre-tested before being administered to actual participants. The pilot study was conducted offline by providing the tool to 20 pharmacy students from each university who were not included in the main study. During pre-testing, students were asked to report any issues with understanding the questions, the sequence of questions, instructions, and the time taken to complete the questionnaire. The items were found to be clear and socially appropriate; no difficulties were observed and hence, no further changes were made.

### Ethical approval

Ethics approvals were attained from both, the Medical Ethics Committees of Xi’an Jiaotong University China (MP202103), and the Ethical Review Board of The Superior University, Lahore. A pre-approval from each student was also obtained in written form on the first page before starting the survey. All participants had to click an agree button before moving to the survey instrument. None of the personal information (name, address phone number etc.) was asked purposefully and participant were assured that the data would only be used for research purposes and treated with confidentiality.

### Data analysis

Cumulative score for the perception and attitude sections were calculated to provide an overall representation of pharmacy students perceptions and attitudes towards pharmaceutical promotion, and pharmaceutical companies as well as incentives. This innovative scoring method was developed based on individual students’ responses. Students were inclined towards promotion and pharmaceutical companies received higher score and vice versa. Some questions were framed in a negative context so a response of strongly disagree yielded the highest score. Responses reflecting positive attitudes towards interactions with medical representatives were assigned a score of 5 for strongly agree and 4 for Agree, Neutral responses received a score of 3, 2 for disagree and 1 for strongly disagree. The cumulative score for each student was then computed. The data were imported into SPSS version 21.0. and cross checked by two researchers for accuracy and completeness.

A simple frequency test was employed for analyzing the demographics and individual questions. The Chi-square test was used (Uncorrected x 2-test and Fisher exact test) was used to evaluate the significance of difference between demographics and other independent variables with each individual item. Dependent variables were calculated in terms of cumulative attitude and perception score. Additionally, an independent t-test and ANOVA were used to compare scores among groups of pharmacy students based on various demographic variables such as gender, year of study, medical school, monthly parental income, parental professions in pharmaceutical or medical fields and other independent variables. In order to check if the total attitude and perception score is continuous/normally distributed we performed normality test before conducting the *t*-test. A significance level of *p* < 0.05 was used to determine the significance of associations in all the aforementioned tests.

## Results

The link was distributed among nearly all students from 3rd to final year but only 1,267 pharmacy students completed the online survey. Out of the total 1,195 surveys were fully completed the questionnaire. The average age was 22.2 ± 1.2 years, approximately 2/3rd of the students were males (62.2%) and almost the same number was from private pharmacy colleges (63.3%). As for parental income, around 58.3% of students had parents earning over 100 K per month (Table 1).

**Table 1:** Demographics information of 1,195 pharmacy students.

No.	Demographic info and statements	No (%)
1	Gender	
	Female	452 (37.8)
	Male	743 (62.2)
2	Age in years (Mean ± SD)	22.2 ± 1.2
3	Year of study in the Pharmacy school	
	3rd Year	412 (34.5)
	4th Year	342 (28.6)
	Final Year	441 (36.9)
4	Institution	
	Private Pharmacy College	757 (63.3)
	Gov Pharrnacy College	438 (36.7)
5	Approximate parental income	
	<PKR 30, 000	151 (12.6)
	PKR 30,000 – PKR 50,000	351 (29.4)
	PKR 50,001 – PKR 100,000	116 (9.7)
	>PKR 100,000	577 (58.3)
6	Have you ever participation in any programs of drug companies?	
	Yes	152 (12.7)
	No	1,043 (87.3)
7	Do you have any parent(s) who is a pharmacist?	
	Yes	212 (17.7)
	No	983 (82.3)
8	Views on current promotional activities.	
	Should be increased	54 (4.5)
	Current level is adequate	326 (27.3)
	Should be decreased	518 (43.3)
	I have no idea	297 (24.9)
9	Does your parent or relative have a community pharmacy shop?	
	Yes	77 (6.4)
	No	1,118 (93.6)
10	Do you have at least one parent working for the pharmaceutical industry?	
	Yes	79 (6.6)
	No	1,116 (93.4)
11	Have you heard about pharmaceutical promotion for drugs?	
	Yes	846 (70.8)
	No	349 (29.2)

Table 2 contains the student counts for the perception items. As given, 27.9% of students agreed that pharmaceutical promotions provided the confidence to counsel patients and an equal number of students stated that promotional material serve as a valuable source of education. Three fifth of (59.8%) of the participants considered it appropriate to accept gifts for educational purposes while 22.6% said that these promotions can lead to unnecessary prescribing or sales of drugs. The responses for each Likert items were aggregated resulting in a mean score of 57.41 ± 5.61 with scores ranging from a minimum of 43 to a maximum of 73 (after inverting the score of negative items).

**Table 2:** Perceptions of 1,195 pharmacy students about pharmaceutical promotion of drugs.

Q No.	Statement	1	2	3	4	5
1	Do you think that pharmacists have to deliver information obtain in pharmaceutical promotion about prescription drugs to patients?	2 (0.2)	75 (6.3)	444 (37.2)	391 (32.7)	283 (23.7)
2	Do you think that pharmaceutical promotion can give pharmacist confidence to counsel patients about their concerns?	12 (1.0)	183 (15.3)	667 (55.8)	208 (17.4)	125 (10.5)
3	The educational activities supported by companies and drug information circular provided good support for education?	25 (2.1)	99 (8.3)	740 (61.9)	266 (22.3)	65 (5.4)
4	The promotional activities of pharmaceutical companies affect physicians’ prescribing practices.?	0 (0.0)	230 (19.2)	490 (41.0)	475 (37.8)	0 (0.0)
5	The promotional activities of pharmaceutical companies affect pharmacist’s dispensing practices?	98 (8.2)	146 (12.2)	456 (38.2)	495 (41.4)	0 (0.0)
6	Do you think that pharmaceutical promotion can promote unnecessary visits to hospitals?	78 (6.5)	250 (20.9)	649 (54.3)	218 (18.2)	0 (0.0)
7	Do you think that pharmaceutical promotion can prevent incorrect information on drugs from being spread?	42 (3.5)	294 (24.6)	621 (52.0)	207 (17.3)	31 (2.6)
8	Do you think that pharmaceutical promotion can restrict pharmacist choices for dispensing of drugs?	0 (0.0)	231 (19.3)	532 (44.5)	390 (32.6)	42 (3.5)
9	Do you expect that pharmaceutical promotion will increase the profits of pharmaceutical companies?	0 (0.0)	94 (7.9)	335 (28.0)	643 (53.8)	123 (10.3)
10	It is ethical for pharmaceutical companies to finance scientific research.?	65 (5.4)	136 (11.4)	596 (49.9)	398 (33.3)	0 (0.0)
11	It is acceptable to participate in the social activities such as dinners arranged by companies	0 (0.0)	278 (23.3)	568 (47.6)	349 (29.2)	0 (0.0)
12	It is appropriate to accept the gifts for educational purposes distributed by companies	160 (13.4)	160 (13.4)	160 (13.4)	357 (29.8)	358 (30.0)
13	I think it is appropriate to accept drug samples given by the companies?	132 (11.0)	302 (25.3)	155 (13.0)	303 (25.4)	303 (25.4)
14	It is appropriate to accept books, journals and other educational material distributed by companies?	120 (10.0)	280 (23.4)	130 (10.9)	384 (32.1)	281 (23.5)
15	It is appropriate to accept the support of the companies to participate in congresses?	81 (6.8)	274 (22.9)	409 (34.2)	274 (22.9)	157 (13.1)
16	Companies do not pass on promotional expenses to drug prices	71 (5.9)	145 (12.1)	541 (45.3)	327 (27.4)	111 (9.3)
17	Company promotions affect the advisory behavior or drug information of pharmacists.	0 (0.0)	219 (18.3)	571 (47.8)	370 (31.0)	35 (2.9)
18	Company promotions do not cause unnecessary prescribing or sales of drugs	27 (2.3)	242 (20.3)	647 (54.1)	217 (18.2)	62 (5.2)

5 = Strongly agree, 4 = Agree, 3 = Neutral, 2 = Disagree, 1 = Strongly disagree.

The mean score of attitude items was 72.20 ± 7.21. The respondents scored a maximum of 92 and a minimum of 52. Almost half (48.7%) of students believed that pharmaceutical promotion is necessary for pharmacist and 46.8% were willing to use this information for future patient counseling. More than half (57.3%) of the respondents proposed that government should ensure the pre-approval of promotion activities and 52.8% thought that students should not meet with medical representatives. Approximately 49.5% of students disagreed that they were adequately taught to handle promotional tactics and representatives while 51.4% expressed dissatisfaction on curriculum regarding education about pharmaceutical promotion.

Regarding the association of the pharmaceutical promotion with drug and antibiotic dispensing practices, 43.9% of students believed that meetings with representatives contribute to irrational drug dispensing, and 49.0% reported that it leads to increased irrational dispensing of antibiotics. Around 55.2% held the view that pharmacists who accept more gifts from companies tend to dispense more antibiotics than others. Alarmingly, 42.1% acknowledged that they might dispense more antibiotics under the influence of gifts from pharmaceutical companies in the future (Table 3).

**Table 3:** Attitudes of 1,195 pharmacy college students about pharmaceutical promotion.

No.	Statement	1	2	3	4	5
1	Do you think that pharmaceutical promotion for drugs is necessary for pharmacist?	2 (0.2)	66 (5.5)	545 (45.6)	326 (27.3)	256 (21.4)
2	Are you willing to actively utilize the data obtained from pharmaceutical promotion when counseling patients in the future?	73 (6.1)	162 (13.6)	412 (34.5)	308 (25.8)	240 (20.1)
3	Are you willing to actively accept patients’ opinions when they ask you to dispense, fill, or administer drugs which they have knowledge due to pharmaceutical promotion in the future?	40 (3.3)	199 (16.7)	578 (48.4)	179 (15.0)	199 (16.7)
4	Do you think that pharmaceutical promotion should not be permitted on the websites of drug companies?	13 (1.1)	198 (16.6)	494 (41.3)	175 (14.6)	315 (26.4)
5	Do you think that pharmaceutical promotion can create unrealistic expectations about drugs?	13 (1.1)	147 (12.3)	475 (39.7)	425 (35.6)	135 (11.3)
6	Do you expect that pharmaceutical promotion for drugs will lead to increasing drug prices due to cost on marketing strategies?	71 (5.9)	145 (12.1)	541 (45.3)	327 (27.4)	111 (9.3)
7	Do you think that the government should mandate preapproval of all pharmaceutical promotion for drugs if they are permitted?	22 (1.8)	202 (16.9)	286 (23.9)	550 (46.0)	135 (11.3)
8	It is unacceptable for a pharmacist to receive a gift from a drug company in any form?	193 (16.2)	425 (35.6)	247 (20.7)	251 (21.0)	79 (6.6)
9	Five drugs from five different companies are identical in terms of price, efficacy and effectiveness. I would preferentially dispense a drug from one of the companies that provided me with such gifts or incentives those from companies that did not.	225 (18.8)	157 (13.1)	334 (27.9)	311 (26.0)	168 (14.1)
10	Pharmacy students should not have any interaction with drug companies in pharmacy school	22 (1.8)	169 (14.1)	254 (21.3)	615 (51.5)	135 (11.3)
11	The information provided about drug effectiveness from pharmaceutical companies is untrustworthy	11 (0.9)	152 (12.7)	637 (53.3)	271 (22.7)	124 (10.4)
12	Do you think you have taught enough about the pharmaceutical promotion handling?	0 (0.0)	592 (49.5)	330 (27.6)	273 (22.8)	0 (0.0)
13	It is acceptable for drug companies to sponsor events/educational seminars during pharmacy school	127 (10.6)	272 (22.8)	398 (33.3)	253 (21.2)	145 (12.1)
14	Do you feel that the syllabus provides you enough knowledge about how to interpret the knowledge given during the promotional activity?	0 (0.0)	614 (51.4)	288 (24.1)	232 (19.4)	61 (5.1)
15	Do you think these interactions between the pharmacist and sales reps should be regularized?	22 (1.8)	168 (14.1)	259 (21.7)	547 (45.8)	199 (16.7)
16	Do you think that the gifts and other things given by pharmaceutical industries to the community pharmacist should be recorded by the gov as in many developed countries?	16 (1.3)	184 (15.4)	271 (22.7)	538 (45.0)	186 (15.6)
17	Do you feel that there is a need for incorporating guidelines regarding relationship between the pharmaceutical industry and the pharmacist in the undergraduate curriculum?	0 (0.0)	122 (10.2)	308 (25.8)	478 (40.0)	287 (24.0)
18	Do you think that pharmaceutical promotion for drugs can have a negative effect on pharmacist’ dispensing practices?	1 (0.1)	204 (17.1)	454 (38.0)	494 (41.3)	42 (3.5)
19	Do you feel that these interactions with representative is one of the key factors in the irrational dispensing of DRUGS?	0 (0.0)	181 (15.1)	490 (41.0)	491 (41.1)	33 (2.8)
20	Do you feel that these interactions with representative is one of the key factors in the irrational dispensing of ANTIBIOTICS?	0 (0.0)	160 (13.4)	450 (37.7)	552 (46.2)	33 (2.8)
21	Do you feel that community pharmacist who meet representatives more often dispense more ANTIBIOTICS?	1 (0.1)	147 (12.3)	426 (32.6)	588 (49.1)	33 (2.8)
22	You may dispense ANTIBIOTICS under the influence of the promotional activity?	10 (0.8)	331 (27.7)	452 (37.8)	365 (30.5)	37 (3.1)
23	Do you feel that those pharmacists who accept more gifts from companies dispense more ANTIBIOTICS than others?	1 (0.1)	146 (12.2)	388 (32.5)	629 (52.6)	31 (2.6)
24	You may dispense ANTIBIOTICS under the influence of acceptance of gifts by pharmaceutical companies?	1 (0.1)	251 (21.0)	440 (36.8)	479 (40.1)	24 (2.0)

5 = Strongly agree, 4 = Agree, 3 = Neutral, 2 = Disagree, 1 = Strongly disagree.

The perception scores were notably higher for final year students, students with parental income are <3,000PKR than other students (*p* < 0.005). These subgroups demonstrated considerably more complaint behavior towards perception items (Table 4).

**Table 4:** Association of perception score (18 items) with demographics values.

No.	Demographic	Mean ± SD	*p*	Eta squared
1	Gender			
	Female	57.11 ± 5.54	0.146	0.002
	Male	57.69 ± 5.72		
2	Year of study in the Pharmacy school			
	3rd Year	56.21 ± 4.89	0.000	0.024
	4th Year	57.87 ± 4.79		
	Final Year	58.17 ± 6.57		
3	Institution			
	Private Pharmacy College	57.54 ± 5.45	0.289	0.002
	Gov Pharmacy College	57.18 ± 5.87		
4	Approximate parental income			
	<PKR 30, 000	58.23 ± 6.26		
	PKR 30,000 – PKR 50,000	57.33 ± 5.65	0.031	0.007
	PKR 50,001 – PKR 100,000	56.19 ± 5.76		
	>PKR 100,000	57.49 ± 5.4		
5	Have you ever participation in any programs of drug companies?			
	Yes	56.86 ± 3.32	0.193	0.001
	No	57.49 ± 5.87		
6	Do you have any parent(s) who is a pharmacist?			
	Yes	57.42 ± 5.72	0.842	0.000
	No	57.34 ± 5.01		
7	Views on current promotional activities.			
	Should be increased	57.63 ± 5.39		
	Current level is adequate	57.27 ± 5.56	0.496	0.002
	Should be decreased	57.66 ± 5.88		
	I have no idea	57.08 ± 5.22		
8	Does your parent or relative have a community pharmacy shop?			
	Yes	57.77 ± 5.0	0.564	0.000
	No	57.38 ± 5.65		
9	Do you have at least one parent working for the pharmaceutical industry?			
	Yes	57.47 ± 5.33	0.923	0.000
	No	57.41 ± 5.66		
10	Have you heard about pharmaceutical promotion for drugs?			
	Yes	57.49 ± 5.58	0.851	0.000
	No	57.30 ± 5.69		

Q7, Q16, Q18 were negative so strongly disagree given 5 and strongly agree given 1 score.

In Table 5 we presented the association of attitude score with various independent variables. Among the demographic’s factors, students in higher school year, those attending government colleges, and those with lower parental income depicted higher compliance towards promotional activities (*p* < 0.005).

**Table 5:** Attitude score association with demographic items of 1,195 pharmacy students.

No.	Demographic association with attitude score (24 items)	Mean ± SD	*p*	Eta squared
1	Gender			
	Female	71.90 ± 7.21	0.251	0.001
	Male	72.4 ± 7.14		
2	Year of study in the pharmacy school			
	3rd Year	69.8 ± 6.68	0.000	0.62
	4th Year	72.84 ± 5.41		
	Final Year	73.95 ± 8.25		
3	Institution			
	Private Pharmacy College	71.72 ± 6.67	0.001	0.008
	Gov Pharmacy College	73.04 ± 8.01		
4	Approximate parental income			
	<PKR 30, 000	75.50 ± 8.1	0.000	0.033
	PKR 30,00 0 – PKR 50,000	71.44 ± 7.00		
	PKR 50,001 – PKR 100,000	70.90 ± 7.02		
	>PKR 100,000	72.00 ± 6.91		
5	Have you ever participation in any programs of drug companies?			
	Yes	71.78 ± 5.20	0.42	0.00
	No	72.26 ± 7.4		
6	Do you have any parent(s) who is a pharmacist?			
	Yes	72.33 ± 6.68	0.000	0.04
	No	70.17 ± 7.32		
7	Views on current promotional activities.			
	Should be increased	72.1 ± 6.6	0.345	0.000
	Current level is adequate	71.9 ± 7.5		
	Should be decreased	72.6 ± 7.3		
	I have no idea	71.7 ± 6.7		
8	Does your parent or relative have a community pharmacy shop?			
	Yes	72.34 ± 7.24	0.08	0.002
	No	71.19 ± 6.55		
9	Do you have at least one parent working for the pharmaceutical industry?			
	Yes	71.25 ± 5.9	0.226	0.000
	No	72.27 ± 7.2		
10	Have you heard about pharmaceutical promotion for drugs?			
	Yes	72.27 ± 7.31	0.000	0.04
	No	70.11 ± 6.91		

Q4, 10, 15,16,17,18 was negatively categorized so strongly disagree given 5 and strongly agree given 1 score.

In our study 14.5% of pharmacy students expressed comfort in accepting the monetary value ranging from 25,000 to 50,000 PKR from pharmaceutical companies while 24.4% of the population agreed to participate in a seminar organized by pharmaceutical company if they were willing to pay over 30 percent of the current year’s fee. Additionally in this study more than two fifth of the (41.9%) students stated that medical representatives are aligned with doctors and the pharmacists and play an important role in the health care system.

## Discussion

In Pakistan pharmacist are typically the last healthcare professionals to interact with patients or caregivers. Moreover, it’s a common practice to dispense medication without requiring a prescription. This situation makes pharmacists susceptible to being targeted by drug companies. Previously there were no studies conducted in Pakistan that examined the impact of pharmaceutical promotion the dispensing practices of pharmacists, with the exception of one study carried out by us (3). The past literature indicates that a physician’s interactions with medical representatives generally commence during medical school (12). Students’ attitudes towards interactions with medical representatives and the gifts offered by pharmaceutical companies revealed the need for strengthening of education and guidance (20). Numerous studies have been conducted to evaluate the impact of pharmaceutical promotion on medical students (8, 12) but none have focused on pharmacy students. To the best of our knowledge this study is the first of its kind to evaluate the perceptions and attitudes of Pharmacy students towards pharmaceutical promotion in Pakistan. The primary findings of this study demonstrated that pharmacy students’ understanding of pharmaceutical promotion and interaction with the pharmaceutical companies was at best unclear. Almost 1/3rd of the students was uncertain about whether they would dispense more antibiotics under the influence of acceptance gifts from pharmaceutical companies with 42.1% agreeing with this statement. This is a deeply concerning situation as it could potentially predict irrational dispensing of antibiotics. Such behavior could initiate a chain reaction leading to an increase in irrational dispensing of antibiotics and subsequently contribute to antimicrobial resistance. This concern was also highlighted in the previous study (21).

Only 12.7% of the respondents have ever participated in training programs conducted by drug companies with the number increasing in senior classes (17.2% in final year vs. 8.0% in 3rd year). This participation rate is notably lower compared to a study conducted in Turkey, where 73% of participants were engaged in similar activity. Likewise, in other studies conducted in Turkey 29.0% of nursing students and 91.2% of medical students were involved in some form of marketing engagement by pharmaceutical companies (22, 23). In our study pharmacy students (24.1%) reported receiving less training regarding ethical issues associated with pharmaceutical promotion and how to interpret promotional material similar to the results observed in the Kuwaiti study (4). There is dire need for proper education of future healthcare professionals (including Pharmacists, nurses and Physicians) to equip them with appropriate decision-making skills for prescribing and dispensing medications as well as to foster ethical relationships with medical representatives (4). However, such essential information is often not provided by most schools and is scarce in in its availability. A comprehensive survey covering 91 Pharmacy and 137 medical schools revealed that teaching about pharmaceutical promotion is severely limited often confined to a single 1-to-2-h lecture or a small assignment activity (24). To effect positive changes in behaviors and attitudes towards pharmaceutical promotion more advanced and innovative teaching techniques are necessary including real time interaction with the medical representative or promotional materials (4). Additionally, it is elaborated that 59.8% of respondents considered it appropriate to accept gifts for educational purposes, 50.8% found it appropriate to accept drug samples from companies, and 53.6% consider it appropriate to accept books, journals and other educational materials distributed by companies. Similar results were obtained from a study in conducted in Turkey (6). Sarikaya et al. in their study highlighted that medical students must be well informed of the societal financial implications of free samples (23). Students also believed that pharmaceutical promotion could impact the advisory behavior of pharmacists, a percentage much lower than what was observed in a similar study conducted in Turkey (6).

According to 13.6% of respondents in the current study, pharmaceutical companies’ information regarding the efficacy of their products is reliable and helpful. This result was in line with a Saudi Arabian study wherein 75% of pharmacists and 65% of physicians said the pharmaceutical companies were a good source of information (25). Other investigations also observed similar findings (26). Nonetheless, some data indicates that overt pharmaceutical company’s advertising might sometimes have unfavorable effects, indicating the need for careful observation. Direct pharmaceutical company marketing has been linked to higher prescribing frequencies, higher costs, or lower quality of prescribing (27). Furthermore, there is a deluge of evidence demonstrating that medical representatives and pharmaceutical companies did not disclose all relevant information about their drugs, particularly when it came to major adverse events (24). According to a survey conducted in France, medical representatives failed to mention negative product effects in around 70% of cases, and about one-third of the advertisements talked about dosages or indications that were not authorized (28).

Regarding the irrational dispensing of drugs and antibiotics, almost an equal number of pharmacists believed that pharmaceutical promotion is key factor contributing to the irrational dispensing of drugs and antibiotics. They also perceived that pharmacists who frequently interact with medical representative tend dispense more antibiotics and 55.2% believed that pharmacists who accept more gifts from companies are more likely to dispense more antibiotics. These results align with a previous study conducted among community pharmacists in Pakistan where 76.2% thought that such promotions could lead to an increase in the irrational dispensing of antibiotics and 18.6% acknowledged dispensing antibiotics due to these promotions (3). The practices are strongly corelated with the behaviors learned during medical school and addressing the chain reaction requires intervention at the foundation level. In our country there is a notable absence of basic ethical codes governing promotion and the relationship between healthcare professionals and the pharmaceutical companies. Furthermore, there is lack of the skills for interacting with medical representatives and this aspect has not been integrated into the pharmacy curriculum. In a previous study the majority of participants expressed their desire to work in community pharmacies, where they can engage in active communication with medical representatives. In a study by Simsek et al., it was found that more than 60% of pharmacy students were planning to establish their own private pharmacy practice (29). A curriculum with proper guidelines is necessary to prepare future pharmacist to play a more impactful role in society (3, 30). In terms of the cumulative perception and attitude scores and their association with demographics a significant correlation was observed with educational years and parental income lower than 30,000PKR. The most probable reason could be the difference in the socio-economic back grounds among students which might lead them to find free opportunities from pharma companies more appealing (12). The study demands implementation of the restrictive policies to arrest the frequency of contact with medical representatives during study period. The implementation of such policies was found the be effective in Canada and was associated with the future behavior of medical students (31).

### Strengths and limitations

Since this was the first study among pharmacy students in Pakistan, it lays a strong foundation by revealing the inclination of pharmacy students towards pharmaceutical promotion and the subsequent need for further research. The non-random snowball selection of pharmacy students introduces the possibility of selection bias, where students with more positive attitudes towards promotion or ethical behavior might have declined to participate. If this were the case, the exposure to promotional activity could be even higher than observed in this study. Additionally, responses might be influenced by recall bias and the results rely on self-reporting rather than measuring actual behavior. As this is a cross-sectional study, it’s challenging to ascertain whether certain factors were causally related to the development of specific behaviors in pharmacy students, However, it provides insight into the prevalence of such behaviors. Further intervention or prospective studies are required to address these issues conclusively. Another potential limitation pertains to the generalizability of results as the study was conducted only in Punjab and across 6 universities. Comprehensive multi center surveys are necessary to obtain a holistic understanding of pharmacy students across the entire country.

## Conclusion

A small number of pharmacy students participated in program by pharmaceutical companies, but a large number of students agreed with the view that promotional activities could influence the attitude of pharmacist and potentially alter the dispensing practices. They also believed that pharmaceutical promotion is contributing to the irrational prescribing of drugs and antibiotics. Interestingly, few students expressed their willingness to dispense more antibiotics due to the influence of promotion. This study emphasized educational requirement for students. It is imperative to appoint educators who can provide training to students on the ethical aspects of relationships between healthcare professionals and pharmaceutical companies within the formal pharmacy curriculum. While, the results, are specific to Pakistan, they might have implications for other countries worldwide suggesting the need to implement similar measures.

## Data Availability

The raw data supporting the conclusions of this article will be made available by the authors, without undue reservation.
