# Supplementary material for: Cross-sectional assessment of perception and attitude of pharmacy students towards pharmaceutical promotion: a study from developing country, Pakistan
Source: Front Med (Lausanne). 2024 Nov 1;11:1424352. doi: 10.3389/fmed.2024.1424352 (PMC11566137; doi:10.3389/fmed.2024.1424352)
Supplement: Supplementary file 1 [file Table_1.docx]

**Demographic association with individual perception.**

| **No** | **Demographics** | **Q1** | **Q2** | **Q3** | **Q4** | **Q5** | **Q6** | **Q7** | **Q8** | **Q9** | **Q10** | **Q11** | **Q12** | **Q13** | **Q14** | **Q15** | **Q16** | **Q17** | **Q18** |
| --- | --- | --- | --- | --- | --- | --- | --- | --- | --- | --- | --- | --- | --- | --- | --- | --- | --- | --- | --- |
| **1** | **Gender** | 0.941 | 0.087 | 0.000 | 0.118 | 0.123 | 0.013 | 0.324 | 0.344 | 0.067 | 0.253 | 0.044 | 0.831 | 0.857 | 0.913 | 0.001 | 0.000 | 0.468 | 0.000 |
| 2 | **Year of study in the Pharmacy school** | 0.000 | 0.000 | 0.000 | 0.000 | 0.000 | 0.000 | 0.000 | 0.000 | 0.000 | 0.000 | 0.000 | 0.567 | 0.005 | 0.001 | 0.000 | 0.000 | 0.000 | 0.000 |
| 3 | **Institution** | 0.488 | 0.000 | 0.000 | 0.000 | 0.000 | 0.025 | 0.000 | 0.000 | 0.000 | 0.019 | 0.056 | 0.911 | 0.119 | 0.259 | 0.001 | 0.000 | 0.001 | 0.000 |
| 4 | **Approximate parental income** | 0.002 | 0.000 | 0.000 | 0.000 | 0.000 | 0.000 | 0.000 | 0.000 | 0.000 | 0.000 | 0.000 | 0.251 | 0.008 | 0.021 | 0.000 | 0.000 | 0.000 | 0.000 |
| **5** | **Have you ever participation in any training programmers of drug companies?** | 0.779 | 0.668 | 0.000 | 0.001 | 0.000 | 0.000 | 0.000 | 0.000 | 0.000 | 0.000 | 0.000 | 0.000 | 0.000 | 0.000 | 0.000 | 0.000 | 0.000 | 0.000 |
| **6** | **Do you have any parent(s) who is a pharmacist?** | 0.802 | 0.204 | 0.173 | 0.484 | 0.850 | 0.665 | 0.373 | 0.084 | 0.132 | 0.548 | 0.074 | 0.837 | 0.685 | 0.289 | 0.628 | 0.848 | 0.017 | 0.930 |
| **7** | **Views on current promotional activities.** | 0.001 | 0.054 | 0.002 | 0.106 | 0.0000 | 0.001 | 0.66 | 0.029 | 0.000 | 0.000 | 0.124 | 0.431 | 0.187 | 0.357 | 0.000 | 0.000 | 0.079 | 0.000 |
| **8** | **Does your parent or relative have a community pharmacy shop?** | 0.717 | 0.506 | 0.679 | 0.045 | 0.098 | 0.046 | 0.620 | 0.801 | 0.931 | 0.473 | 0.988 | 0.976 | 0.442 | 0.081 | 0.908 | 0.425 | 0.702 | 0.728 |
| **9** | **Do you have at least one parent working for the pharmaceutical industry?** | 0.032 | 0.200 | 0.944 | 0.820 | 0.965 | 0.860 | 0.169 | 0.685 | 0.506 | 0.541 | 0.918 | 0.316 | 0.660 | 0.511 | 0.603 | 0.084 | 0.313 | 0.950 |
| **10** | **Have you heard about pharmaceutical promotion for drugs?** | 0.555 | 0.011 | 0.000 | 0.512 | 0.002 | 0.000 | 0.162 | 0.022 | 0.001 | 0.000 | 0.057 | 0.717 | 0.161 | 0.100 | 0.597 | 0.000 | 0.029 | 0.001 |

**Chi square test = P value <0.05 considered significant**
