# Supplementary material for: Cross-sectional assessment of perception and attitude of pharmacy students towards pharmaceutical promotion: a study from developing country, Pakistan
Source: Front Med (Lausanne). 2024 Nov 1;11:1424352. doi: 10.3389/fmed.2024.1424352 (PMC11566137; doi:10.3389/fmed.2024.1424352)
Supplement: Supplementary file 2 [file Table_2.docx]

**Demographic association with individual attitude items.**

| **No** | **Demographics** | **Q1** | **Q2** | **Q3** | **Q4** | **Q5** | **Q6** | **Q7** | **Q8** | **Q9** | **Q10** | **Q11** | **Q12** | **Q13** | **Q14** | **Q15** | **Q16** | **Q17** | **Q18** |
| --- | --- | --- | --- | --- | --- | --- | --- | --- | --- | --- | --- | --- | --- | --- | --- | --- | --- | --- | --- |
| **1** | **Gender** | 0.850 | 0.003 | 0.002 | 0.026 | 0.002 | 0.001 | 0.194 | 0.564 | 0.932 | 0.499 | 0.076 | 0.591 | 0.001 | 0.105 | 0.273 | 0.742 | 0.737 | 0.903 |
| 2 | **Year of study in the Pharmacy school** | 0.000 | 0.000 | 0.000 | 0.000 | 0.000 | 0.000 | 0.000 | 0.000 | 0.000 | 0.000 | 0.000 | 0.000 | 0.000 | 0.000 | 0.000 | 0.000 | 0.000 | 0.000 |
| 3 | **Institution** | 0.581 | 0.003 | 0.002 | 0.001 | 0.000 | 0.000 | 0.000 | 0.000 | 0.687 | 0.846 | 0.000 | 0.000 | 0.000 | 0.198 | 0.006 | 0.211 | 0.000 | 0.000 |
| 4 | **Approximate parental income** | 0.001 | 0.000 | 0.000 | 0.000 | 0.000 | 0.000 | 0.000 | 0.000 | 0.000 | 0.000 | 0.000 | 0.000 | 0.000 | 0.000 | 0.000 | 0.000 | 0.000 | 0.000 |
| **5** | **Have you ever participation in any training programmers of drug companies?** | 0.073 | 0.000 | 0.000 | 0.000 | 0.000 | 0.000 | 0.000 | 0.000 | 0.000 | 0.000 | 0.724 | 0.060 | 0.000 | 0.000 | 0.000 | 0.000 | 0.000 | 0.000 |
| **6** | **Do you have any parent(s) who is a pharmacist?** | 0.292 | 0.251 | 0.231 | 0.300 | 0.538 | 0.848 | 0.294 | 0.937 | 0.838 | 0.456 | 0.201 | 0.961 | 0.726 | 0.930 | 0.433 | 0.817 | 0.831 | 0.309 |
| **7** | **Views on current promotional activities.** | 0.000 | 0.000 | 0.000 | 0.128 | 0.022 | 0.000 | 0.009 | 0.000 | 0.000 | 0.055 | 0.050 | 0.000 | 0.000 | 0.000 | 0.000 | 0.000 | 0.000 | 0.014 |
| **8** | **Does your parent or relative have a community pharmacy shop?** | 0.676 | 0.332 | 0.447 | 0.726 | 0.565 | 0.425 | 0.865 | 0.990 | 0.599 | 0.946 | 0.649 | 0.718 | 0.749 | 0.943 | 0.918 | 0.887 | 0.145 | 0.941 |
| **9** | **Do you have at least one parent working for the pharmaceutical industry?** | 0.055 | 0.550 | 0.905 | 0.524 | 0.890 | 0.084 | 0.263 | 0.404 | 0.544 | 0.402 | 0.117 | 0.262 | 0.866 | 0.841 | 0.218 | 0.317 | 0.516 | 0.569 |
| **10** | **Have you heard about pharmaceutical promotion for drugs?** | 0.646 | 0.046 | 0.003 | 0.291 | 0.563 | 0.000 | 0.444 | 0.014 | 0.146 | 0.333 | 0.023 | 0.005 | 0.394 | 0.000 | 0.170 | 0.017 | 0.449 | 0.047 |
| **No** | **Demographics** | **Q19** | **Q20** | **Q21** | **Q22** | **Q23** | **Q24** |  |  |  |  |  |  |  |  |  |  |  |  |
| **1** | **Gender** | 0.371 | 0.149 | 0.136 | 0.690 | 0.260 | 0.323 |  |  |  |  |  |  |  |  |  |  |  |  |
| 2 | **Year of study in the Pharmacy school** | 0.000 | 0.000 | 0.000 | 0.000 | 0.000 | 0.000 |  |  |  |  |  |  |  |  |  |  |  |  |
| 3 | **Institution** | 0.317 | 0.001 | 0.000 | 0.000 | 0.001 | 0.000 |  |  |  |  |  |  |  |  |  |  |  |  |
| 4 | **Approximate parental income** | 0.000 | 0.000 | 0.000 | 0.000 | 0.000 | 0.000 |  |  |  |  |  |  |  |  |  |  |  |  |
| **5** | **Have you ever participation in any training programmers of drug companies?** | 0.000 | 0.000 | 0.000 | 0.000 | 0.000 | 0.000 |  |  |  |  |  |  |  |  |  |  |  |  |
| **6** | **Do you have any parent(s) who is a pharmacist?** | 0.135 | 0.438 | 0.147 | 0.379 | 0.183 | 0.202 |  |  |  |  |  |  |  |  |  |  |  |  |
| **7** | **Views on current promotional activities.** | 0.079 | 0.029 | 0.034 | 0.001 | 0.071 | 0.167 |  |  |  |  |  |  |  |  |  |  |  |  |
| **8** | **Does your parent or relative have a community pharmacy shop?** | 0.868 | 0.875 | 0.806 | 0.678 | 0.829 | 0.971 |  |  |  |  |  |  |  |  |  |  |  |  |
| **9** | **Do you have at least one parent working for the pharmaceutical industry?** | 0.441 | 0.624 | 0.590 | 0.582 | 0.808 | 0.863 |  |  |  |  |  |  |  |  |  |  |  |  |
| **10** | **Have you heard about pharmaceutical promotion for drugs?** | 0.300 | 0.271 | 0.445 | 0.046 | 0.329 | 0.251 |  |  |  |  |  |  |  |  |  |  |  |  |

**Chi square test = P value <0.05 considered significant**
