# Supplementary material for: Cross-sectional assessment of perception and attitude of pharmacy students towards pharmaceutical promotion: a study from developing country, Pakistan
Source: Front Med (Lausanne). 2024 Nov 1;11:1424352. doi: 10.3389/fmed.2024.1424352 (PMC11566137; doi:10.3389/fmed.2024.1424352)
Supplement: Supplementary file 3 [file Data_Sheet_1.docx]

**Attitudes of pharmacy students towards incentives offered by pharmaceutical companies- perspective from a Pakistan- a cross-sectional study.**

**Serial ID____________**

**Name (Optional)____________**

**Demographics**

1. **Sex**
2. Male
3. Female
4. Age in years_________
5. **Year of study in the Pharmacy School**
6. Year 3
7. Year 4
8. Year 5
9. **Institution**
10. Private Pharmacy College
11. Government Pharmacy College
12. **Approximate parental income (monthly)**
13. < PKR 30, 000
14. PKR 30,001 – PKR 50,000
15. PKR50,001 – PKR 100,000
16. > PKR 100,000
17. **Career Plan?**

Self Employed Community Pharmacist.

Industrial Pharmacist

Government Job (hospital DTL, etc)

Academic pharmacist

Other________

1. **Have you ever Participation in any training programmers of drug companies**

Yes

No

1. **Do you have any parent(s) who is a pharmacist?**
2. Yes
3. No
4. **Views on current promotional activities**

Should be Increased

Current level is adequate

Should be decreased

I have no idea

1. **Does your parent or relative have a Community Pharmacy shop?**

Yes

No

1. **Do you have at least one parent working for the pharmaceutical industry?**
2. Yes
3. No
4. **What is your current health status?**
5. Poor 2) Acceptable 3) Good 4) Very good
6. **Have you heard about pharmaceutical promotion for prescription drugs?**
7. Yes 2) No
8. **Have you heard about direct-to-consumer advertising (DTCA) for prescription drugs?**
9. Yes 2) No

**Perceptions of pharmacy students about pharmaceutical promotion of drugs.**

**5 = Strongly agree, 4 = Agree, 3 = Neutral, 2 = Disagree, 1 = Strongly disagree**

| **No** | **Statement** | **5** | **4** | **3** | **2** | **1** |
| --- | --- | --- | --- | --- | --- | --- |
| 1 | Do you think that pharmacists have to deliver information about prescription drugs to patients? |  |  |  |  |  |
| 2 | Do you think that Pharmaceutical Promotion can give pharmacist confidence to counsel patients about their concerns? |  |  |  |  |  |
| 3 | The educational activities supported by companies and drug information circular provided good support for education. |  |  |  |  |  |
| 4 | The promotional activities of pharmaceutical companies affect physicians’ prescribing practices. |  |  |  |  |  |
| 5 | The promotional activities of pharmaceutical companies affect pharmacist dispensing practices |  |  |  |  |  |
| 6 | Do you think that Pharmaceutical Promotion can promote unnecessary visits to hospitals? |  |  |  |  |  |
| 7 | Do you think that Pharmaceutical Promotion can prevent incorrect information on drugs from being spread? |  |  |  |  |  |
| 8 | Do you think that Pharmaceutical Promotion can restrict pharmacist choices for dispensing of drugs? |  |  |  |  |  |
| 9 | Do you expect that Pharmaceutical Promotion will increase the profits of pharmaceutical companies? |  |  |  |  |  |
| 10 | It is ethical for pharmaceutical companies to finance scientific research. |  |  |  |  |  |
| 11 | It is acceptable to participate in the social activities such as dinners arranged by companies |  |  |  |  |  |
| 12 | It is appropriate to accept the gifts for educational purposes distributed by companies |  |  |  |  |  |
| 13 | I think it is appropriate to accept drug samples given by the companies |  |  |  |  |  |
| 14 | It is appropriate to accept books, journals and other educational material distributed by companies |  |  |  |  |  |
| 15 | It is appropriate to accept the support of the companies to participate in congresses |  |  |  |  |  |
| 16 | Companies do not pass on promotional expenses to drug prices |  |  |  |  |  |
| 17 | Company promotions affect the advisory behavior or drug information of pharmacists |  |  |  |  |  |
| 18 | Company promotions do not cause unnecessary prescribing or sales of drugs |  |  |  |  |  |

**Attitudes of Pharmacy college students about pharmaceutical promotion.**

**5 = Strongly agree, 4 = Agree, 3 = Neutral, 2 = Disagree, 1 = Strongly disagree**

| **No** | **Statement** | **5** | **4** | **3** | **2** | **1** |
| --- | --- | --- | --- | --- | --- | --- |
| 1 | Do you think that Pharmaceutical Promotion for drugs is necessary for Pharmacist? |  |  |  |  |  |
| 2 | Are you willing to actively utilize the data obtained from Pharmaceutical Promotion when counselling patients in the future? |  |  |  |  |  |
| 3 | Are you willing to actively accept patients’ opinions when they ask you to dispense, fill, or administer drugs which they have knowledge due to Pharmaceutical Promotion in the future? |  |  |  |  |  |
| 4 | Do you think that Pharmaceutical Promotion should not be permitted on the websites of drug companies? |  |  |  |  |  |
| 5 | Do you think that Pharmaceutical Promotion can create unrealistic expectations about drugs? |  |  |  |  |  |
| 6 | Do you expect that Pharmaceutical Promotion for drugs will lead to increasing drug prices due to cost on marketing strategies? |  |  |  |  |  |
| 7 | Do you think that the government should mandate preapproval of all Pharmaceutical Promotion for drugs if they are permitted? |  |  |  |  |  |
| 8 | It is unacceptable for a pharmacist to receive a gift from a drug company in any form |  |  |  |  |  |
| 9 | Five drugs from five different companies are identical in terms of price, efficacy and effectiveness. I would preferentially dispense a drug from one of the companies that provided me with such gifts or incentives those from companies that did not. |  |  |  |  |  |
| 10 | Pharmacy students should not have any interaction with drug companies in pharmacy school |  |  |  |  |  |
| 11 | The information provided about drug effectiveness from pharmaceutical companies is untrustworthy |  |  |  |  |  |
| 12 | Do you think you have taught enough about the pharmaceutical promotion handling? |  |  |  |  |  |
| 13 | It is acceptable for drug companies to sponsor events/educational seminars during pharmacy school |  |  |  |  |  |
| 14 | Do you feel that the syllabus provides you enough knowledge about how to interpret the knowledge given during the promotional activity? |  |  |  |  |  |
| 15 | Do you think these interactions between the Pharmacist and sales reps should be regularized? |  |  |  |  |  |
| 16 | Do you think that the gifts and other things given by pharmaceutical industries to the Community Pharmacist should be recorded by the gov as in many developed countries? |  |  |  |  |  |
| 17 | Do you feel that there is a need for incorporating guidelines regarding relationship between the pharmaceutical industry and the Pharmacist in the undergraduate curriculum? |  |  |  |  |  |
| 18 | Do you think that Pharmaceutical Promotion for drugs can have a negative effect on pharmacist’ dispensing practices? |  |  |  |  |  |
| 19 | Do you feel that these interactions with representative is one of the key factors in the irrational dispensing of drugs? |  |  |  |  |  |
| 20 | Do you feel that these interactions with representative is one of the key factors in the irrational dispensing of antibiotics? |  |  |  |  |  |
| 21 | Do you feel that community pharmacist who meet representatives more often dispense more antibiotics? |  |  |  |  |  |
| 22 | Will you dispense more antibiotics under the influence of the promotional activity? |  |  |  |  |  |
| 23 | Do you feel that those pharmacists who accept more gifts from companies dispense more antibiotics than others? |  |  |  |  |  |
| 24 | Will you dispense more antibiotics under the influence of acceptance of Gifts by pharmaceutical companies? |  |  |  |  |  |

**Questions related to Attitude**

1. **I would feel comfortable accepting gifts from a pharmaceutical company worth**
2. < PKR 2500
3. PKR 2500 - 5000
4. PKR 5000 - 25000
5. PKR 25000 - 50000
6. > PKR 50000
7. **A drug company wants to increase its visibility to the community pharmacy and has recently approached the Pharmacy school. They would like to provide a one-day seminar regarding their product at the end of the year. In return, they are willing to pay for a fraction of the years tuition for each student who attends their seminar. As a pharmacy student faced with increasing tuition costs, I think that it would be fair if the pharmaceutical company pays this percentage of my year’s pharmacy school tuition:**
8. 1 – 10% b. 10 – 20% c. 20 - 30% d. more than 30%
9. **Which of the following statements do you think is most true of drug companies:**
10. They are fundamentally on the same side as doctors and patients and should be regarded as an important part of the health care system.
11. They are fundamentally interested in profit and never on the side of wither doctors or patients.
12. They are primarily interested in profit: however, they still try to work in the best interest of doctors and patients.
13. **Briefly, what are your views about interactions between pharmacy students and the pharmaceutical company?**

______________________________________________________________________________

______________________________________________________________________________

1. **Have you had any interactions with the pharmaceutical industry in the past that might have influenced your responses to the above questions? If yes please specify:**

______________________________________________________________________________

______________________________________________________________________________
